# Subfoveal Choroidal Thickness after Panretinal Photocoagulation with Red and Green Laser in Bilateral Proliferative Diabetic Retinopathy Patients: Short Term Results

**DOI:** 10.1155/2016/9364861

**Published:** 2016-08-09

**Authors:** Ramak Roohipoor, Sina Dantism, Aliasghar Ahmadraji, Reza Karkhaneh, Mohammad Zarei, Fariba Ghasemi

**Affiliations:** Eye Research Center, Farabi Eye Hospital, Tehran University of Medical Science, Tehran, Iran

## Abstract

*Purpose*. To compare subfoveal choroidal, central retinal, and peripapillary nerve fiber layer (RNFL) thickness after panretinal photocoagulation (PRP) with red and green laser in diabetic patients.* Study Design*. Randomized clinical trial.* Methods*. A total of 50 patients with bilateral proliferative diabetic retinopathy and no diabetic macular edema underwent PRP. One eye was randomly assigned to red or green laser. Subfoveal choroidal, central retinal, and RNFL thicknesses were evaluated at baseline and 6 weeks after treatment.* Results*. The mean subfoveal choroidal, central retinal, and peripapillary nerve fiber layer (RNFL) thickness increased significantly in each eye 6 weeks after PRP (*P* values in red laser group: <0.01, 0.03, and <0.01, resp., and in green laser group <0.01, <0.01, and <0.01). There was no difference between red and green laser considering subfoveal choroidal, central retinal, and peripapillary nerve fiber layer (RNFL) thickness increase after PRP (*P* values: 0.184, 0.404, and 0.726, resp.).* Conclusion*. Both red and green lasers increased mean subfoveal choroidal, central retinal, and peripapillary nerve fiber layer (RNFL) thickness significantly 6 weeks after PRP, but there is no difference between these two modalities in this regard.

## 1. Introduction

Diabetic retinopathy is one of the most common causes of visual loss worldwide. According to Early Treatment of Diabetic Retinopathy Study (ETDRS), protocol panretinal photocoagulation (PRP) can prevent severe visual loss in proliferative diabetic retinopathy (PDR) [1]. Several studies have shown histopathologic changes in choroid of diabetic patients [2–4]. Meanwhile, using panretinal photocoagulation (PRP), as was suggested beneficially by diabetic retinopathy study for proliferative diabetic retinopathy, has gotten general acceptance [1]. With the advent of enhanced depth imaging optical coherence tomography (EDI-OCT), it is now possible to visualize and measure choroidal changes easily.

Two different laser types for PRP are common, red and green laser. To the best of our knowledge, no other study has compared these two laser modalities in changing choroidal or central retinal thickness. In this study, we have evaluated the subfoveal choroidal, central retinal, and peripapillary retinal nerve fiber layer (RNFL) thickness changes before and after PRP using EDI-OCT and we compare the green and red laser in this regard.

## 2. Methods

This clinical trial was approved by the Institutional Review Board/Ethics Committee. Written informed consents were obtained from all participants. Eligible cases were 50 patients (100 eyes) with bilateral proliferative diabetic retinopathy. Exclusion criteria were advanced proliferative diabetic retinopathy, a history of any laser treatment (panretinal or focal laser photocoagulation), any history of intravitreal drug injection, ocular surgery, significant media opacity, myopia, or hyperopia with refractive error of more than 3 diopters.

Primary objective was to determine subfoveal choroidal and retinal thickness and peripapillary nerve fiber layer thickness changes 6 weeks after red versus PRP laser.

A complete ophthalmic examination including best corrected visual acuity (BCVA) using Snellen chart, Goldmann applanation tonometry, and dilated indirect ophthalmoscopy was conducted. Diabetic retinopathy grading (DRG) was performed according to the ETDRS definition [5]. Enhanced depth imaging optical coherence tomography (EDI-OCT) mapping was performed using SD-OCT (Spectralis®, Heidelberg Engineering, Heidelberg, Germany). All patients were treated on an outpatient basis. PRP was performed in 3 sessions with one week interval. A total number of 1600 to 2000 spots per eye were applied to ablate retina using Ellex Integre Pro Scan laser photocoagulator (82 Gilbert Street, Adelaide, SA 5000, Australia). The order of treated areas was as follows: nasal, inferior, superior, and then temporal retina. Time exposure was 100 milliseconds. To have threshold laser photocoagulation, power setting was chosen to have a mild gray-white burn (Grade 2) according to ETDRS guidelines [1]. Laser setting parameters did not differ significantly between two groups (mean power 303 mWatts in red and 290 mWatts in green laser group, *P* value: 0.14; mean laser spots 1789 spots in red and 1869 spots in green laser group, *P* value: 0.13).

One eye of all patients was randomly assigned to red laser and the other eye to green laser. Spectral domain optical coherence tomography images were obtained using Spectralis OCT (Heidelberg Engineering, Heidelberg, Germany) to measure central retinal thickness and enhanced depth imaging (EDI) mode to measure subfoveal choroidal thickness. BCVA measurement and an OCT scan were performed at baseline and 6 weeks after completion of PRP. Subfoveal choroidal thickness was measured using apparatus measurement tool at baseline and 6 weeks after PRP. Choroidal segmentation was done manually, moving the reference lines from the retinal borders to the choroidal borders. The internal limiting membrane line was moved to the base of the retinal pigment epithelium. The basement membrane line was moved to chorioscleral junction. Also retinal nerve fiber layer (RNFL) thickness analysis was done at baseline and 6 weeks after treatment.

### 2.1. Statistical Analysis

Data were analyzed using SPSS software (version 16, SPSS, Inc.). Data normality was assessed using Shapiro-Wilk test. A paired sample *t*-test was used to compare macula and choroid thickness before and after treatment. Independent sample *t*-test was performed to compare macula and choroid thickness changes from baseline between two groups. General Linear Model was applied to assess differences within and between group. *P* values of <0.05 were considered significant.

## 3. Results

A total number of 50 patients with PDR entered the study. The mean age of the patients ± standard deviation was 55.6 ± 8.9 years. Nineteen patients were male (38%). The mean visual acuity of patients was 0.32 ± 0.28 logMAR and 0.27 ± 0.23 logMAR in red laser group and green laser group, respectively. The mean HbA1C was 8.47 ± 1.57%. The baseline characteristics of the patients are presented in Table 1. No complication occurred in both groups during laser and in follow-up period.

The mean subfoveal CT increased significantly in each group at 6 weeks follow-up (in red laser group, 202.14 ± 24.5 micron at baseline and 211.7 ± 26.6 micron at 6 weeks, *P* value < 0.00, showing 4.86% CT increase compared to baseline) (in green group, 201.9 ± 21.13 micron at baseline and 208.9 ± 25.0 at 6 weeks, *P*  value < 0.00, showing 3.54% increase compared to baseline) (Table 2).

There was not a significant difference between red and green laser in terms of choroidal thickness changes from baseline (*P* value 0.184).

In red laser group, the mean central retinal thickness was 271.6 ± 30.33 micron at baseline and 298.5 ± 62.14 micron at week 6 of follow-up (*P* value 0.03). In green laser group, the mean central retinal thickness was 267.0 ± 36.9 micron at baseline and 306.4 ± 73.3 micron at week 6 of follow-up, (*P* value 0.000 for both groups). There was no significant difference between macular thickness increase between two groups (*P* value: 0.404) (Table 2).

There was no significant association between choroidal thickness change and retinal thickness change in each group (*P* values: 0.051 and 0.52, resp.).

The mean peripapillary RNFL thickness was 100.5 ± 20.1 micron in red laser group at baseline which increased to 108.2 ± 23.1 micron at 6 weeks (*P* value 0.000) (7.8% as compared to baseline). In green laser group, it was 103.5 ± 19.8 micron at baseline which increased to 112.7 ± 22.9 in week 6 of follow-up (*P* value 0.000) (9.2% as compared to baseline). RNFL change between two groups was not significant (*P* value: 0.762) (Table 2).

## 4. Discussion

In this study, in eyes with proliferative diabetic retinopathy and without significant macular edema, the mean subfoveal choroidal, central retinal, and peripapillary RNFL thickness increased significantly after both red and green argon laser treatments.

This is in accordance with some of previously published studies that confirm this finding [6–9]. Takahashi et al. measured changes in choroidal blood flow in the foveal area one month after PRP in patients with severe diabetic retinopathy and no macular edema using a laser-Doppler flowmetry. They reported that PRP increases both the choroidal blood flow and the choroidal blood volume. Also, Cho et al. in their case series reported that PRP induced increases in both SFCT and macular thickness. Changes in subfoveal choroidal thickness did not correlate with changes in macular thickness in their study.

Two possible mechanisms are proposed by which this increase occurs. First, it has been attributed to redistribution of choroidal blood flow [10–14]. After PRP, following degradation of photoreceptors, blood flow of treated areas decreases. This leads to redistribution of blood in other untreated areas, especially the macula. Following blood flow increase, choroidal thickness increases in the macular area. This redistribution mechanism is documented in animals in some prior studies [10–17]. The second hypothesis is the inflammation triggered by PRP. This transient inflammation probably causes release of cytokines and leads to increase of blood flow and choroidal thickness [18, 19].

Mean CT increase in both groups in this study is similar to what have been reported before in other trials [7–10]. It is noteworthy that, in some other trials, the measured subfoveal CT showed decreased thickness after PRP in short term follow-up. They have hypothesized that the reason may be due to the destruction of choroidal vasculature after thermal damage of PRP [20, 21].

Also, we found that central macular thickness and RNFL thickness increased significantly in both groups; this is also similar to what has been found in other studies [20–24]. This finding is similar to what has been reported before about the increase of retinal thickness after PRP and it was attributed to the release of proinflammatory cytokines or hypoxia-induced macular edema [20–24].

Also, we did not find any correlation between changes of choroidal thickness and central retinal thickness. This is similar to what has been reported by Cho et al. [7].

One of the novel analyses done in this study is the comparison between red and green laser in changing retinal, choroidal, and RNFL thickness. Although all three measured variables showed significant increase after treatment in each eye, intereye comparisons were not statistically significant considering central retinal, subfoveal choroidal, and RNFL thickness changes (*P* values: 0.404, 0.184, and 0.762, resp.) that may indicate no considerable difference between red and green laser in this regard. To the best of our knowledge, there is only one report by Ghassemi et al. [24] that compared the difference between red and green laser in PDR patients. They reported significant increase of RNFL thickness after PRP without any difference between red and green laser. This is in accordance with our findings in this study.

One of the advantages of this study is selection of both eyes of one patient which can exclude the effect of systemic factors on retinal, choroidal, and RNFL thickness.

This study has some limitations including low sample size and short follow-up time. Nonetheless, this study showed significant increase of subfoveal choroidal, foveal retinal, and peripapillary RNFL thickness after red and green laser PRP and no significant difference between these two lasers in aforementioned measurements.

## Figures and Tables

**Table 1:** Baseline characteristics.

Parameter	Statistics
Age	55.6 ± 8.9 years
Sex	38% male (19 patients), 62% female (31 patients)
HbA1C	8.47 ± 1.57%
VA	0.32 ± 0.28 logMAR in red group and 0.27 ± 0.23 logMAR in green group

**Table 2:** Measured thicknesses at baseline and week 6 of follow-up.

	Baseline	6 weeks	*P* value (within each group)	*P* value (between red and green laser group)
Subfoveal choroidal thickness (red laser group)	202.14 ± 24.5	211.7 ± 26.6	<0.000	0.184
Subfoveal choroidal thickness (green laser group)	201.9 ± 21.13	208.9 ± 25.0	<0.000	0.184
Central retinal thickness (red laser group)	271.6 ± 30.33	298.5 ± 62.14	0.03	0.404
Central retinal thickness (green laser group)	267.0 ± 36.9	306.4 ± 73.3	<0.000	0.404
RNFL thickness (red laser group)	100.5 ± 20.1	108.2 ± 23.1	<0.000	0.726
RNFL thickness (green laser group)	103.5 ± 19.	112.7 ± 22.9	<0.000	0.726
